# Comparison of Aroma Compounds and Sensory Characteristics between Two Different Types of Rice-Based Baijiu

**DOI:** 10.3390/foods13050681

**Published:** 2024-02-23

**Authors:** Fan Wu, Shaohui Fan, Guoliang He, Siyu Liang, Yan Xu, Ke Tang

**Affiliations:** 1 Lab of Brewing Microbiology and Applied Enzymology, School of Biotechnology, Jiangnan University, 1800 Lihu Avenue, Wuxi 214122, China; fanwu123@foxmail.com (F.W.); biosean@126.com (Y.X.); 2 Key Laboratory of Industrial Biotechnology of Ministry of Education, State Key Laboratory of Food Science and Technology, Jiangnan University, 1800 Lihu Avenue, Wuxi 214122, China; 3 Guangdong Shiwan Baijiu Group Company Ltd., Foshan 528000, China; fanshaohui6688@163.com (S.F.); heguoliang2023@163.com (G.H.); liangsiyu8866@163.com (S.L.)

**Keywords:** rice-based Baijiu, sensory evaluation, aroma, GC-O-MS, odor activity values (OAVs), multivariate statistical analysis

## Abstract

Rice-based Baijiu has gained popularity in the Chinese market. Qingya-flavored Baijiu, a variant of Xiaoqu-fermented Baijiu, employs rice as its primary raw material, with an improved production process compared to traditional rice-flavored Baijiu. We comprehensively characterized and compared the aroma profiles of these two rice-based Baijiu types using static sensory experiments (QDA, quantitative descriptive analysis) and dynamic sensory experiments (TDS, temporal dominance of sensations). Qingya-flavored Baijiu exhibited pronounced plant, oily, and roasted aromas, while traditional rice-flavored Baijiu displayed more prominent fruity, floral, and sour notes. Utilizing GC-O-MS (gas chromatography-olfactometry–mass spectrometry) and multi-method quantification, we qualitatively and quantitatively analyzed 61 key aroma compounds, identifying 22 compounds with significant aroma contributions based on odor activity values (OAVs). Statistical analyses, combining sensory and chemical results, were conducted to predict important aroma compounds responsible for the aroma differences between the two Baijiu types. Aroma Recombination and Omission experiments showed that seven compounds play key roles in the aroma of Qingya-flavored Baijiu, including (2E,4E)-Deca-2,4-dienal, linalool, apricolin, ethyl acetate, ethyl isobutyrate, ethyl caprylate, and ethyl isovalerate.

## 1. Introduction

**Figure 1:**
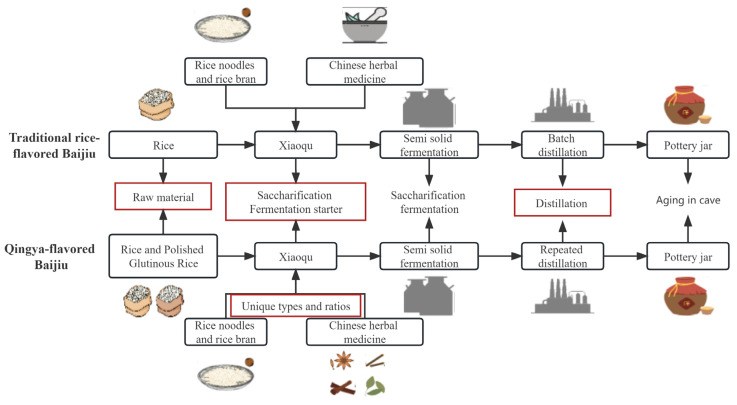
Brief process flow diagram. The steps in the red boxes are main different brewing processes between two types of Baijiu.

Baijiu, a traditional distilled spirit made through the fermentation and distillation of grains, offers a rich history, and occupies a significant position in the global spirits market [1,2]. Since ancient times, rice has had a very important position in China; ancient China’s most popular alcoholic beverages are mainly rice as raw materials. At present in the southern half of China, rice-based Baijiu is still the mainstream choice. The aroma of Baijiu significantly influences consumers’ decision-making processes [3]. To cater to diverse market preferences, manufacturers have introduced innovative techniques to produce Baijiu with distinct aroma profiles, building upon traditional production methods and creating a range of unique and enticing aroma characteristics. Traditional rice-flavored Baijiu is manufactured utilizing rice as the primary ingredient, along with Xiaoqu as the saccharifying agent [4]. The production process involves semi-solid fermentation, followed by distillation, aging, and blending. Building upon the brewing process of traditional rice-flavored Baijiu, Qingya-flavored Baijiu incorporates glutinous rice into the raw materials. Additionally, it selects wild strains from nature and introduces licorice, cinnamon leaves, and other medicinal and edible Chinese herbs to create Xiaoqu (Figure 1). Kettle distillation is employed during the distillation phase, and specific caves are chosen for the aging process, resulting in the production of Qingya-flavored Baijiu with a distinct and unique flavor.

Aroma volatile compounds represent the key constituents contributing to food aroma, exerting a significant influence on the flavor and quality of Baijiu [5]. The collective content of aroma compounds in Baijiu constitutes less than 2% of the overall composition [6]. Currently, more than 2000 aroma volatile compounds have been identified in Baijiu, yet only a subset of these compounds plays a pivotal role in shaping the aroma profile of Baijiu [7,8]. To better quantify and ensure product quality, it is crucial to identify the key aroma compounds in various Baijiu types. Research on the aroma of rice-flavored Baijiu remains relatively limited, with most studies comparing it to other distilled liquors with similar raw materials. Yin et al. [9] compared the characteristics of compounds in rice-flavored Baijiu, Japanese awamori, and kome shochu, three spirits with similar brewing processes. They found that the solid saccharification method may result in rice-flavored Baijiu containing a substantial amount of short-chain acid ethyl esters, such as ethyl isobutyrate and ethyl lactate, along with medium-chain fatty acid ethyl esters, with ethyl lactate being a key aroma compound unique to rice-flavored Baijiu. In addition, to search for aroma-active compounds more directly and accurately, GC-O combined with GC-MS (GC-O-MS, gas chromatography-olfactometry–mass spectrometry), known for its unique advantages in qualitative aroma compound analysis, was introduced to Baijiu aroma compound research. Fan et al. [2] identified odor active compounds in Wuliangye using normal-phase fractionation and GC-O, and detected the most important aroma compounds in Wuliangye and Jiannanchun liquors using aroma extract dilution analysis (AEDA). Fan et al. [10] identified odor-active compounds in Chixiang-flavored Baijiu through GC-O and aroma extract dilution analysis (AEDA), and further verified important aroma volatiles according to OAV.

Chemical analysis methods are limited to compound detection and cannot provide a comprehensive sensory description of Baijiu. Hence, sensory experiments are essential in Baijiu aroma studies. Currently, QDA (quantitative descriptive analysis) is the most widely used method in the food flavor field. This experiment allows for precise descriptions of aroma attributes and quantifies their intensity [11,12]. Zhao [13] used QDA to distinguish aroma differences between rice-flavored Baijiu and awamori, elucidating the static sensory characteristics of rice-flavored Baijiu with mellow, fruity, and floral aromas. While QDA can elucidate the sensory characteristics of liquor in the glass, it cannot accurately describe changes in aroma attributes during Baijiu consumption. TDS (temporal dominance of sensations) can compensate for this limitation. However, the application of TDS in Baijiu aroma research remains limited. Combining QDA and TDS allows for a more comprehensive characterization of the overall aroma characteristics of Baijiu and its aroma differences compared to other Baijiu [14].

Due to the lack of research on important aroma compounds in rice-based Baijiu, the quality of its characteristic aroma can only depend on experience. Therefore, this study aims to elucidate flavor differences between two kinds of typical rice-based Baijiu, Qingya-flavored Baijiu (QFB) and traditional rice-flavored Baijiu (RFB), through QDA and TDS. Key aroma compounds were identified using GC-O-MS, accurately quantified through a multi-method approach, and OAVs (odor activity values) and PLSR (partial least squares regression) were employed to predict the aroma compounds contributing to the flavor differences between the two Baijiu types. Finally, the important aroma active compounds were verified by aroma recombination and omission experiments.

## 2. Materials and Methods

### 2.1. Baijiu Samples

Two Qingya-flavored Baijiu and four traditional rice-flavored Baijiu samples were selected, categorizing them into two groups based on alcohol content. Qingya-flavored Baijiu is innovated on the basis of traditional rice-flavored Baijiu production technology. Due to the uniqueness of brewing technology, there is only one enterprise producing it in China at present. These six samples were selected from 17 kinds of rice-made Baijiu by inviting China national Baijiu judges to evaluate. They both have typical characteristics of two types. Table 1 provides specific sample information. All six liquors were stored at 4 °C in a refrigerator.

### 2.2. Chemicals

Chromatographic-grade (GC grade, ≥98%) analytical standards for chemical analyses were used. Ethyl acetate, ethyl isobutyrate, ethyl butyrate, ethyl 2-methylbutanoate, ethyl isovalerate, ethyl valerate, isoamyl acetate, ethyl hexanoate, ethyl lactate, ethyl oenanthate, ethyl caprylate, ethyl nonanoate, ethyl DL-leucate, ethyl benzoate, 3-methylbutyl octanoate, diethyl succinate, ethyl undecanoate, ethyl phenylacetate, phenylethyl acetate, ethyl laurate, ethyl 3-phenylpropanoate, ethyl tetradecanoate, 1-propanol, isobutanol, butanol, 3-methyl-1-butanol, 1-hexanol, 3-octenol, 1-heptanol, 1,1-Diethoxyethane, hexanal, (2E)-2-Nonenal, (2E,4E)-Deca-2,4-dienal, (2E)-2-Octenal, trans-2-Undecen-1-al, (2E)-2-Decenal, nonanal, 2-heptanone, acetoin, 2-nonanone, 3-octen-2-one, linalool, cis-anethol, trans-caryophyllene, citronellol, geranylacetone, acetic acid, isobutyric acid, 2-methylbutyric acid, octanoic acid, 2-amylfuran, furfural, styrene, acetophenone, benzyl alcohol, phenethyl alcohol, apricolin, 1-hexanoic acid, propionic acid, benzaldehyde, 4-ethylphenol were commercially obtained from Sigma-Aldrich Co., Ltd. (Shanghai, China), J&K Scientific Co., Ltd. (Shanghai, China), and Alfa Aesar (Tianjin, China). All internal standards used in quantitative analysis, such as octyl propionate, menthol, pentyl acetate, and pivalic acid, were sourced from Sigma-Aldrich (Shanghai, China).

NaCl and Na_2_SO_4_ were acquired from China National Pharmaceutical Group Corp (Shanghai, China). A mixture of C6-C30 hydrocarbons, ethanol (HPLC grade), and dichloromethane (CH_2_Cl_2_, HPLC grade) was obtained from Titan Scientific Co., Ltd. (Shanghai, China). Ultrapure water was generated using a Milli-Q purification system (Millipore, Bedford, MA, USA).

The QDA sensory references were of chromatographic grade, while the retronasal aroma sensory references were of food grade. Mouth rinsing was performed using purified water from Wahaha Group Co., Ltd., (Hangzhou, China).

### 2.3. Identification of Aroma-Active Compounds

#### 2.3.1. Aroma Extraction by LLE (Liquid–Liquid Extraction)

Following the procedure outlined earlier [14], we extracted the mixture of the sample (50 mL) and saturated saline (200 mL) with CH_2_Cl_2_ (3 × 50 mL) three times. Anhydrous sodium sulfate was added to remove any remaining water. The combined organic layer was placed in a −20 °C refrigerator overnight and then concentrated to 500 µL using nitrogen blowing.

GC-O-MS analysis was conducted using an Agilent 7890B gas chromatograph, an Agilent 5977B mass spectrometer detector, and a sniffing port (ODP 4, Gerstel, Württemberg, Germany). The ODP temperature was 230 °C. Fused silica columns, either an Agilent DB-FFAP (60 m × 0.25 mm × 0.25 μm) or Agilent DB-5 (30 m × 0.32 mm × 0.50 μm) column were used.

Following the protocol described earlier [12], 1 µL of the sample was injected. The gas chromatographic heating program was initiated at an initial temperature of 50 °C, held for 2 min, then increased to 230 °C at a rate of 5 °C·min^−1^, and maintained for 15 min. Helium, with a purity exceeding 99.9999%, was used as the carrier gas at a flow rate of 2 mL·min^−1^. The ion source was EI, operating at 230 °C, with an electron energy of 70 eV. Mass scanning ranged from *m*/*z* 35 to 500, with an 8 min solvent delay time.

OSME (odor-specific magnitude estimation) is a method for evaluating the contribution of aroma compounds based on odor intensity [15]. Three panelists, including one male and two females from Jiangnan University, were selected for GC-O analysis after undergoing sensory training. A 5-point scale was used to assess aroma intensity, with 1 indicating “very weak”, 3 indicating “accurate identification”, and 5 indicating “very strong”. Each panelist evaluated each sample three times, and an odor was confirmed when identified six times. The identification of each aroma compound was determined by comparing it with the retention index (RI), aroma descriptors, and standard products.

#### 2.3.2. Quantitation of Aroma-Active Compounds

Experiments were designed based on the existing literature [10,12]. Different compounds exhibited variations in their response efficiency to various quantitative methods. Highly abundant compounds were quantified using GC-FID (gas chromatography with flame ionization detection), while most aroma compounds were quantified using HS-SPME-GC-MS (headspace solid-phase microextraction-gas chromatography–mass spectrometry). This is because compounds with excessive concentration exhibit overloading in GC-MS, which affects the formulation of the standard curve. In addition, for acidic compounds, the HS-SPME-GC-MS sampling method yielded unstable results, leading us to choose LLME-GC-MS (liquid–liquid microextraction) for quantifying acid aroma compounds. The content of alkenal is low and easily masked in GC-MS, which affects the quantitative accuracy. So, their concentration was quantified using HS-SPME-GC×GC-TOFMS (two-dimensional gas chromatography–time-of-flight mass spectrometry). The specific quantitative methods for different compounds are detailed in Table S1. Standard curves were established by using the ratio of the peak area of the target compound to the internal standard as the X-axis and the ratio of the concentration of the target compound to the internal standard as the Y-axis. To ensure accuracy, three replicates were conducted for each sample and recorded the average of the three measurements.

##### GC-FID

Of the sample, 1 µL was injected using a split injection ratio of 1:10. Internal standards tert-amyl alcohol (final concentration: 111.04 mg/L) and pentyl acetate (final concentration: 125.5 mg/L) were employed. Helium, with a purity exceeding 99.9995%, served as the carrier gas at a flow rate of 1 mL·min^−1^. Additionally, hydrogen flowed at 40 mL·min^−1^. The initial temperature was 50 °C, held for 3 min, then increased gradually to 110 °C at a rate of 6 °C min^−1^. Finally, the temperature was raised to a maximum of 200 °C at a rate of 30 °C min^−1^ and maintained at the highest temperature for 1 min.

##### HS-SPME-GC-MS

The sample was diluted with ultrapure water to an alcohol content of 10%vol. A mixture of 5 mL of the diluted sample and 1.5 g of NaCl was placed in a 20 mL headspace bottle. For quantification, internal standards octyl propionate (final concentration: 119.11 µg/L) and menthol (final concentration: 90 µg/L) were used. We employed the MPS2 system for HS-SPME autosampling. The sample was equilibrated for 5 min at 50 °C with a rotation speed of 400 r·min^−1^ before a 45 min extraction. GC-MS conditions are the same as described in Section 2.3.1.

##### LLME-GC-MS

We diluted the sample with ultrapure water to an alcohol content of 10% vol (total volume 20 mL), added 6 g NaCl, and added 2.5 mL redistilled ether three times for extraction. The quantitative internal standard was 10 µL pivalic acid (11,590 mg/L). Anhydrous sodium sulfate was added to remove residual water. The organic layer was placed in a −20 °C refrigerator overnight and concentrated to 250 µL using nitrogen blowing. The injection volume was 1 µL, GC-MS conditions were the same as in Section 2.3.1.

##### HS-SPME-GC×GC-TOFMS

The literature was referenced and slight modifications were made to the operating conditions [16]. The method of HS-SPME is the same as Section HS-SPME-GC-MS. The equipment was mainly about the 1st (DB-FFAP: 60 m × 0.25 mm × 0.25 μm) and 2nd (Rxi-17Sil MS: 1.5 m × 0.25 mm × 0.25μm) column, automatic sampling device (MPS2), and solid-phase extraction device (Visiprep DL SPE).

The chromatographic conditions were as follows: High-purity helium (purity ≥ 99.9995%) served as the carrier gas at a flow rate of 1 mL/min, with the sample operation set to constant flow mode. The one-dimensional oven’s initial temperature was 45 °C and, after 3 min, increased to 150 °C at a rate of 4 °C/min, maintaining the temperature for 2 min. Subsequently, the temperature continued to rise to 200 °C at a rate of 6 °C/min, and finally, reached 230 °C at a rate of 10 °C/min, remaining at this temperature for 10 min. The second oven’s temperature was maintained 5 °C higher than the first oven’s temperature. The modulation compensation temperature was 20 °C. The modulation period was 4 S (the thermal pulse time was 0.8 s).

The time-of-flight mass spectrometry conditions were as follows: EI ionization source, ion source voltage of 70 eV, ion source temperature of 230 °C, and line temperature of 240 °C. Mass acquisition ranged from *m*/*z* 35 to 400, with a frequency of 100 spectra/s. The detector voltage was 1430 V.

The internal standard for quantitative use is 3-Methyl-d3-thiopanal (final concentration: 51.53 µg/L).

### 2.4. Sensory Evaluation

Experiments were designed based on the existing literature, with appropriate modifications made [14,17].

#### 2.4.1. Panel Selection and Training

A sensory panel recruited from Jiangnan University was utilized in this study. Panelists underwent a screening process to identify sensory defects and undesirable habits, such as smoking. Furthermore, they were required to demonstrate a genuine interest in Baijiu tasting for the purpose of sensory training. Out of the total pool of participants, 30 individuals were selected to partake in the sensory training program. This training regimen spanned a duration of two months and included 16 sessions held twice weekly. The training curriculum was designed to cover various facets of sensory analysis, including the recognition of Baijiu aromas, difference testing, and experimentation with classification scales.

To assess the sensory panel’s performance, measures of sensory repeatability, discrimination, and overall consistency were conducted employing Panelcheck (MATFORSK). Subsequent to the completion of the training program, 22 individuals were chosen from the initial group of 30 to serve as panelists in the subsequent phases of the experiment. Panelists received compensation for both their participation in the training sessions and their involvement in the formal experiments.

#### 2.4.2. QDA

After a one-hour sensory meeting, eight sensory descriptors were selected: flower, sweet, fruity, rice, plant, roasted, sour, and oily aroma. Specific sensory descriptors and their references can be found in Table S2. By referring to the literature and combining with the opinions of officials, the concentrations of reference substances and compounds were set. Sensory panelists were instructed to score the sensory descriptors on a 15 cm line scale ranging from “0 = not present” on the left end to “9 = very intense” on the right end of the scale. In order to foster greater agreement among the panelists concerning sensory descriptors and intensity evaluation, the outcomes from the preceding session were shared with them. Consequently, eight sensory panelists who demonstrated exceptional stability, repeatability, and consistency were selected to partake in the formal experiment. The tasting procedure commenced with the distribution of each sample to the panelists, and these samples were presented in a randomized sequence. Panelists proceeded to assess the intensities of various predetermined attributes utilizing a 15 cm line scale ranging from 0 to 9.

#### 2.4.3. TDS

After a single session, eight specific sensory descriptors were identified, namely, sweetness, stimulation, bitterness, fruity aroma, rice aroma, plant aroma, roasted aroma, and oily aroma. The detailed descriptions and references for each of these descriptors are given in Table S2. Except for the oily aroma, which used the Baijiu sample as the reference, other senses used the specific compound solution or real object as the reference.

The SensoMaker tool was employed to conduct dynamic sensory experiments within the oral cavity. To mitigate the potential influence of the descriptor order on sensory perceptions, the sequence in which descriptors were presented to individual panelists was varied. Each panelist was presented with six samples, each containing 2 mL and labeled with a random three-digit number. Each sample underwent a 90 s evaluation period, commencing upon entry into the mouth and concluding with swallowing at the 5-S mark. Starting from the moment of entry, panelists were tasked with identifying the most prominent sensory descriptor in the mouth until all sensations dissipated. It is advisable to observe a 5 min intermission between the evaluations of two different samples and to rinse the mouth with clean water during this interval.

### 2.5. Aroma Recombination and Omission Experiments

Q40 was used as a representative sample, compounds with OAVs > 1 were added to the simulated liquor (40% vol) according to their occurring concentrations (Table S2). The aroma characteristics of the reconstructed sample were scored by 8 panelists; the concrete method is consistent with Section 2.4.2. The compounds were divided into 17 groups according to aroma characteristics, structure characteristics, and standardized coefficients. Triangular tests with forced choice were used, which asked panelists to select one cup with a deletion of one or a group of compounds from the three reconstructed samples. The protocol of omission test is the same as Table 4.

### 2.6. Statistical Analysis

SPSS 25.0 (SPSS, Inc., Chicago, IL, USA) was used for analysis of variance (ANOVA). Partial least squares regression (PLSR) analysis was conducted using XLSTAT 2016 (Addinsoft, Paris, France). The TDS curves were generated using SensoMaker software 1.91 (UFLA, Lavras, MG, Brasil).

## 3. Results and Discussion

### 3.1. Sensory Evaluation

#### 3.1.1. QDA (Quantitative Descriptive Analysis)

**Figure 2:**
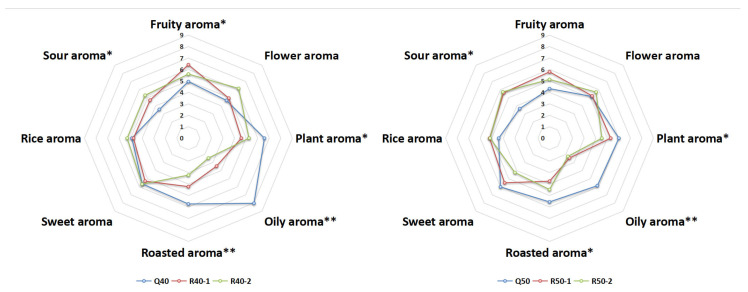
Aroma profile difference between Qingya-flavored and traditional rice-flavored Baijiu. *, significant (*p* ≤ 0.05). **, significant (*p* ≤ 0.01).

The results of QDA were used to construct aroma contour maps for six Baijiu (Figure 2). It was evident that RFB in both groups were quite similar. However, the two QFBs stood out as being distinctly different from RFB in terms of multiple aroma attributes. Specifically, RFB had more advantages in fruity, sour, and rice aroma, while QFB were characterized by dominant aroma attributes such as oily, plant, and roasted aromas.

#### 3.1.2. TDS (Temporal Dominance of Sensations)

Static sensory analysis methods cannot illustrate sensory changes during the drinking process, so the TDS experiment was utilized to clarify the characteristics of flavor changes during drinking. After an hour-long sensory group meeting, five aroma descriptors were selected, including plant aroma, roasted aroma, rice aroma, fruity aroma, and oily aroma. Additionally, sweetness, bitterness, and stimulation were added. These five aroma descriptors were consistent with the specific concepts and references in the static sense. To ensure panelists fully understood these sensory descriptors, definitions and references were established for each descriptor (Table S2).

**Figure 3:**
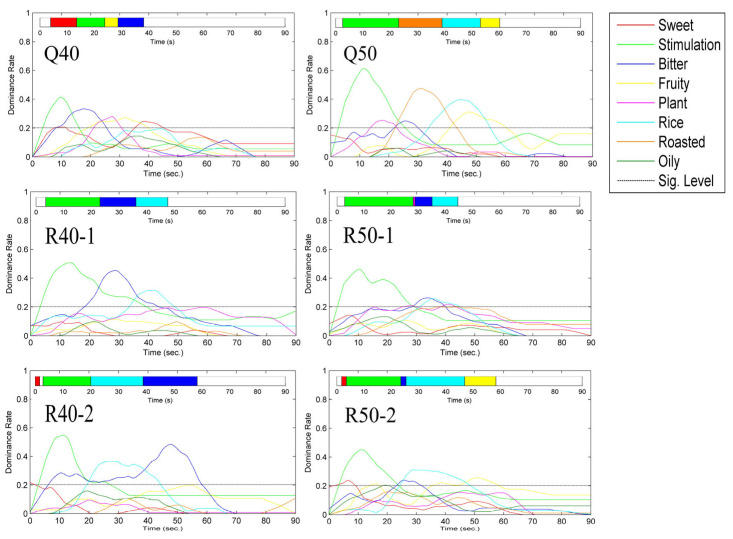
TDS curves of Qingya-flavored and traditional rice-flavored Baijiu.

The TDS curve revealed the primary sensory characteristics during the Baijiu drinking process (Figure 3). Due to the higher ethanol content, all six Baijiu exhibited a longer period of stimulation during the entrance stage. Compared with RFB, QFB displayed a wider variety of aromas, with plant aroma appearing only in Q40, and roasted aroma only in Q50. Among the six Baijiu, only Q50 did not exhibit bitterness, possibly because the TDS results focus more on the most prominent sensory aspects, which may overshadow the bitterness. In general, the higher the alcohol content, the shorter the duration of bitterness and the longer the duration of stimulation. The sweetness was observed in only Q40, R40-2, and R50-2, with R40-2 and R50-2 exhibiting sweetness at the beginning of drinking, while the sweetness of Q40 appeared at the end of drinking.

### 3.2. Identification of Aroma-Active Compounds

Table 2 displays the results obtained through the employment of GC-O in conjunction with GC-MS, which collectively identified 63 aroma compounds within the six samples analyzed. Among these two QFBs, a total of 21 aroma compounds exhibited OSME values surpassing 3, and these compounds are presumed to significantly contribute to the overall aroma profile of the Baijiu. This set of compounds comprises four esters, five acids, three carbonyl compounds, four aromatic compounds, four other compounds, and one unidentified compound. In contrast, RFB contained 20 compounds with OSME values greater than 3, predominantly comprising 5 esters, 5 acids, 4 aromatic compounds, 5 other compounds, and one unidentified compound.

Remarkably, 18 compounds possessing OSME values exceeding 3 were common to both QFB and RFB. This suggests a notable similarity in the chemical composition of the two Baijiu types, making it challenging to clearly distinguish between them based solely on OSME values. Further research endeavors are warranted to unveil the chemical underpinnings for the distinctions observed between the two Baijiu variants.

Additionally, in addition to the identified compounds, two compounds were present at such low concentrations that only their retention times, aroma descriptors, and intensity in the mass spectrum could be recorded. These two compounds contributed to the aroma profile associated with baking and cooked vegetables, respectively.

### 3.3. Quantitation of Aroma-Active Compounds

To gain a better understanding of the aroma compounds that significantly contribute to the overall scent of the two types of Baijiu, we determined the concentrations of 61 aroma compounds detected during the olfactory process. These compounds included 20 esters, 7 alcohols, 5 terpenes, 6 acids, 12 carbonyl compounds (aldehydes and ketones), 8 aromatic compounds, and 3 other compounds.

Different compounds possess varying structural differences, impacting the quantitative efficiency of different methods. Therefore, a range of methods was selected to quantify different types of compounds [18]. The quantitative results for various compounds can be found in Table S3, along with specific information about the standard curve. To identify compounds with significant differences between QFB and RFB within the same group, a variance analysis was conducted for the accurately quantified compounds in all three Baijiu groups. The results revealed 39 compounds with significant differences between Q40 and the two RFB (R40-1 and R40-2), and 34 compounds with significant differences between Q50 and the two RFB (R50-1 and R50-2). Furthermore, 27 compounds exhibited significant differences in both groups, suggesting that these compounds may be the key components responsible for the aroma differences between QFB and RFB. The compounds with significant differences can be found in Table S3.

#### 3.3.1. Esters

Previous research has established that esters play a pivotal role in imparting fruity, floral, and other pleasing aromas to Baijiu [18]. Significantly higher total ester concentrations were observed in RFB samples (R40-2 and R50-2) compared to the other four Baijiu variants. In contrast, there were no substantial differences in the total ester concentrations among the remaining four Baijiu samples. Notably, a detailed analysis of variance unveiled notable variations in the concentrations of eight esters between QFB and RFB.

These eight esters comprised ethyl isobutyrate, ethyl valerate, ethyl isovalerate, ethyl nonanoate, ethyl 2-methylbutanoate, ethyl benzoate, ethyl oenanthate, and ethyl undecanoate. It is noteworthy that these ester compounds in terms of concentration contribute significantly to the flavor diversity observed in the 12 Baijiu types [19].

Specifically, Q40 and Q50 showed higher concentrations of ethyl valerate, ethyl nonanoate, ethyl benzoate, and ethyl oenanthate. On the other hand, RFB exhibited elevated concentrations of ethyl isobutyrate, ethyl isovalerate, and ethyl 2-methylbutanoate. Furthermore, the differences in both the type and concentration of these esters may be the primary factor responsible for the distinct fruity and floral aroma attributes observed in QFB and RFB.

#### 3.3.2. Alcohols

In QFB, notably higher concentrations of 1-propanol, 1-hexanol, and 1-heptanol were observed. These alcohols are primarily associated with green, plant, and fresh aromas. 1-hexanol is a critical aroma compound in strong-flavored Baijiu, contributing to green, fruity, and fatty aromas [20]. Additionally, 3-methyl-1-butanol and 1-hexanol contribute to the bitterness in Baijiu [21].

#### 3.3.3. Aldehydes and Ketones

Baijiu contains carbonyl compounds originating from various sources, such as alcohol oxidation, amino acid deamination, and decarboxylation metabolic pathways [22]. These compounds have lower boiling points compared to alcohols and phenols with the same carbon number, making them more volatile. Due to their high volatility, carbonyl compounds assist in the volatilization of other volatile compounds, ultimately enhancing the overall aroma. Concentration-based analysis of variance revealed significant differences in the concentrations of six acetals and two ketones among the six Baijiu. Specifically, the concentrations of hexanal, nonanal, (2E)-2-Nonenal, (2E,4E)-Deca-2,4-dienal, and 3-octen-2-one were notably higher in the two QFBs. Hexanal emits a green and fatty aroma, while 3-octen-2-one is primarily associated with a nutty aroma. Hexanal is derived from linoleic acid in rice through non-enzymatic or other methods [23], and the difference in hexanal concentration between QFB and RFB can be attributed to the different raw materials. Hexanal contributes to a fresh grass aroma, which may enhance the plant aroma of QFB. (2E)-2-Nonenal, (2E,4E)-Deca-2,4-dienal, and (2E)-2-Decenal were found to be important compounds responsible for the fatty aroma of Chi-flavored Baijiu [10].

#### 3.3.4. Acids

Organic acids play a pivotal role in shaping the aroma quality of Baijiu, as they serve as essential aroma compounds and precursors for ester aroma compounds [24]. Notably, the quantification of organic acids revealed a marked distinction, particularly regarding 1-hexanoic acid. Both QFB variants exhibited higher concentrations of 1-hexanoic acid, which contributes to a refreshing sour and cheese-like flavor. This acid has been scientifically demonstrated to enhance the overall aroma profile [24].

#### 3.3.5. Others

In QFB, several compounds, including cis-anethol, benzyl alcohol, styrene, benzaldehyde, apricolin, and 2-amylfuran, were found in higher concentrations compared to RFB. Research has suggested that benzyl alcohol contributes to the floral aroma in Baijiu [12]. Apricolin is a lactone with a notably low odor detection threshold, contributing to fruity aromas in various Baijiu types. It has also been reported to impart nutty aromas in other distilled spirits [25]. Cis-anethol, when smelled, is characterized by a sweet and pleasant fragrance. It has been detected in medicinal-flavored Baijiu. The variations in cis-anethol concentrations may stem from the medicinal materials incorporated into the Xiaoqu used in the two Baijiu types. Phenethyl alcohol, recognized for its floral and sweet fragrances, constitutes an essential aroma compound in alcoholic beverages [26]. Its concentration in RFB exceeded that in QFB, with the concentration primarily influenced by the level of amino acids present in the mash.

The concentration of 2-amylfuran in QFB surpassed that in RFB of equivalent alcohol content. Notably, 2-amylfuran has been previously identified in cooked rice from California long-grain and brown rice varieties [27]. Personnel have associated it with the distinctive flavor of rice koji [23]. Furfural is generated during the distillation process as a result of monosaccharide degradation under high-temperature and acidic conditions. Consequently, it is frequently detected in Baijiu [28]. Having a furan-like aroma, furfural was found in higher concentrations in the two QFBs compared to RFB, potentially due to variations in the acidic conditions within the fermentation mash of the two Baijiu types. Notably, both furfural and 2-pentylfuran possess a roasted-like aroma, which could contribute to the roasted aroma characteristic of QFB.

### 3.4. OAVs

Concentration alone does not necessarily dictate the impact of a compound on the overall aroma. To better evaluate the influence of compounds, researchers use odor active values (OAVs), calculated for each compound using its threshold value. It is widely accepted that compounds with OAVs greater than 1 are likely to have a noticeable impact on the overall aroma. The OAVs of each compound are shown in Table 3. Among the six Baijiu, 15 compounds had OAVs greater than 1, including 10 esters, 4 alcohols, and 1 aldehyde. Additionally, the two QFBs contained 22 compounds with OAVs greater than 1, while RFB had 16 such compounds.

The OAV of hexanal in RFB was notably lower than in QFB, suggesting its crucial role in creating the distinctive plant aroma of QFB. In RFB, four compounds stood out with OAVs greater than 30, including ethyl caprylate, ethyl isobutyrate, ethyl Dl-leucate, and ethyl isovalerate. Interestingly, the OAVs of these compounds in the two QFBs were significantly lower than in the two RFB. Therefore, they may play a crucial role in contributing to the fruity aroma of RFB. The OAVs of (2E,4E)-Deca-2,4-dienal in the two QFBs was generally greater than 10, but in RFB, the OAVs were significantly smaller. These compounds have a fatty aroma, which may be essential for the outstanding oily aroma in QFB.

### 3.5. Prediction of Potentially Important Aroma Compounds

**Figure 4:**
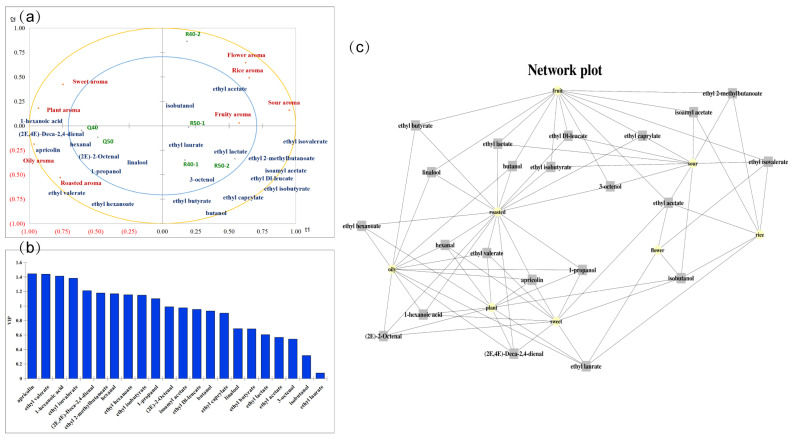
(**a**) Correlation loading plot between the important aroma compounds (OAVs > 1) and aroma attributes. (**b**) Variable importance for the projection values for aroma compounds (OAVs > 1). (**c**) Standardized coefficients between the important aroma compounds (OAVs > 1) and aroma attributes. The two projects connected have positive prediction results.

To predict the aroma compounds that significantly contribute to the characteristic aroma in the two types of Baijiu, a relationship analysis was performed between 8 aroma attributes and the concentrations of 22 compounds with OAVs greater than 1 using partial least squares regression (PLSR) [32]. The Q_2_, R_2_X, and R_2_Y values of both dimensions exceeded 0.4, indicating the reliability of the prediction model. The two types of Baijiu were separated into distinct categories on either side of the X-axis (Figure 4a).

Attributes such as rice, sour, fruity, and floral aromas were closer to RFB, while attributes like plant, oily, roasted, and sweet aromas were closer to QFB. Compounds such as ethyl hexanoate, ethyl valerate, 1-propanol, hexanal, (2E,4E)-Deca-2,4-dienal, (2E)-2-Octenal, (2E)-2-Decenal, 1-hexanoic acid, linalool, and apricolin closely resembled the oily, plant, and roasted aromas. These compounds are likely to be important contributors to these three aromas. 1-propanol, hexanal, (2E,4E)-Deca-2,4-dienal, and (2E)-2-Octenal are also close to the two QFBs, which may have a positive impact on their aroma. Ethyl lactate, ethyl acetate, ethyl isobutyrate, ethyl Dl-leucate, ethyl laurate, and ethyl 2-methylbutanoate were associated with fruity aroma. Among the compounds with OAVs > 1, 10 compounds were found to have a variable importance in projection (VIP) > 1 (Figure 4b), including ethyl valerate, apricolin, 1-hexanoic acid, ethyl isovalerate, ethyl 2-methylbutanoate, (2E,4E)-Deca-2,4-dienal, ethyl isobutyrate, ethyl hexanoate, hexanal, and 1-propanol. These compounds are considered potential aroma compounds contributing to the differences between the two types of Baijiu.

To further assess the correlation between individual compounds and each sensory attribute, standardized coefficients were introduced, focusing on compounds with positive values for plotting (Figure 4c). The standardized coefficients can represent the relationship between the dependent variable and the independent variable, predicting the actual impact of a single compound on the overall aroma. The compounds with a connecting line between the sensory attributes in the graph have a positive impact on that sensory attribute in the prediction (standardized coefficients > 0). The combination of VIP and standardized coefficients indicated that ethyl isobutyrate, ethyl isovalerate, and ethyl 2-methylbutanoate contributed positively to a fruity aroma, which always has high concentrations in RFBs. Compounds that were significantly different predicted to have a positive effect on plant aroma included ethyl hexanoate, ethyl valerate, 1-propanol, hexanal, 1-hexanoic acid, and apricolin. Although the aroma characteristics of some compounds, such as 1-propanol and hexanal, are not directly related to plant aroma, their concentrations are often positively correlated with the intensity of plant aroma, so they may enhance plant aroma through synergistic effects and other side effects. Compounds positively correlated with plant, oily, and roasted aromas showed high repeatability, suggesting a potential synergy between these three aromas. These predictions are based on the relationship between the OAVs of the compound and the intensity of the sensory attribute, and further experiments are needed to verify the sensory contribution of each compound.

### 3.6. Aroma Recombination and Omission Experiments

**Figure 5:**
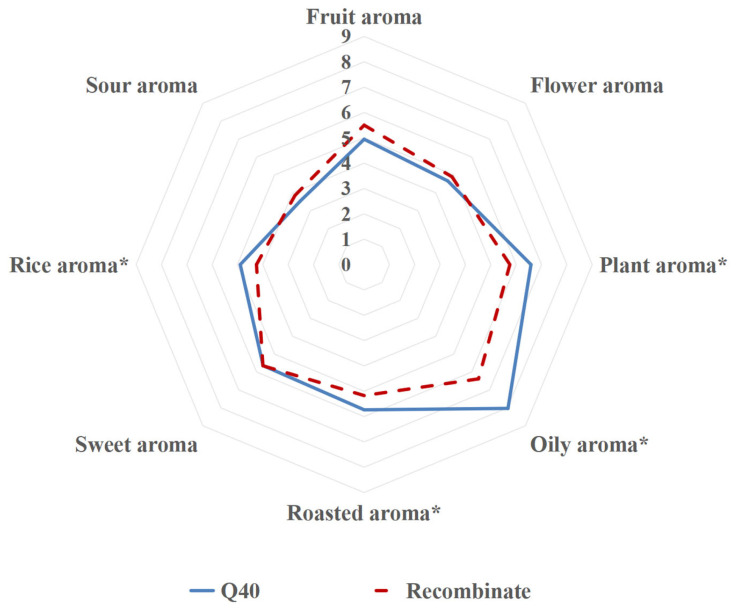
Aroma profile of Qingya-flavored Baijiu (Q40) and the complete aroma recombinate. * Sensory attributes with significant differences.

In order to prove the contribution of the compounds to the aroma of Qingya-flavored Baijiu, the compounds with the concentration above the threshold and the VIPs > 1 in PLSR were added to the alcohol solution (40% vol); the sensory similarities and differences between the samples were analyzed by eight well-trained panelists. As Figure 5 shows, although the eight sensory attributes of the reconstructed samples were relatively similar to those of Q40, there were still significant differences in oily, plant, rice, and roasted aromas. These results indicate that there are some defects in the current methods for the identification of all the important aroma compounds of Qingya-flavored Baijiu, which need to be further explored by other methods.

In order to further judge the effect of known compounds on the aroma of Qingya-flavored Baijiu, 20 groups of compounds were divided according to their aroma characteristics, structural characteristics, and standardized coefficients, and the omission experiments were conducted. Table 4 shows the sensory analysis results of 27 recombination models.

**Table 4:** Results of omission experiments.

No.	Odorants Omitted from the Complete Recombinate	Num ^a^	Significance ^b^
1	all esters	18	***
1-1	ethyl acetate	16	**
1-2	ethyl isobutyrate	15	*
1-3	ethyl caprylate	15	*
1-4	ethyl isovalerate	14	*
1-5	ethyl lactate	8	
1-6	ethyl DL-leucate	10	
1-7	ethyl butyrate	12	
1-8	ethyl 2-methylbutanoate	10	
1-9	ethyl hexanoate	9	
1-10	ethyl valerate	12	
2	all alcohols	13	
3	linalool	14	*
4	apricolin	19	***
5	all aldehydes	27	***
5-1	hexanal	13	
5-2	(2E)-2-Octenal	8	
5-3	(2E,4E)-Deca-2,4-dienal	15	*
6	fatty acids	13	
7	3-octenol	7	

^a^ Number of correct judgments from 27 panelists distinguish the omission models. ^b^ ***, very highly significant (α ≤ 0.001); **, highly significant (α ≤ 0.01); *, significant (α ≤ 0.05).

According to Table 1, the three models separately without all esters (model 1), aldehydes (model 5), and apricolin (model 4) were evaluated with very high significance (α ≤ 0.001). Among them, all panelists can identify model 5 without all aldehydes. This shows that aldehydes play a very important role in the overall aroma of Qingya-flavored Baijiu. However, when aldehydes were deleted one by one, only models 5-3 without (2E,4E)-Deca-2,4-dienal was evaluated with significance (α ≤ 0.05), but not hexanal and (2E)-2-Octenal. These results indicate that (2E,4E)-Deca-2,4-dienal plays a key role in the typical aroma of Qingya-flavored Baijiu which may make an important contribution to the oily aroma, while other aldehydes may have some synergistic effects.

Therefore, the distillation of Qingya-flavored Baijiu may be the reason for the difference between the two types of flavor Baijiu. The results showed that apricolin was sweet, and the absence of apricolin (model 4) was detected significantly, which indicated that apricolin played a key role in the aroma of Qingya-flavored Baijiu and might be the main source of sweet aroma. In the esters, the ethyl acetate (model 1-1), ethyl isobutyrate (model 1-2), ethyl caprylate (model 1-3), and ethyl isovalerate (model 1-4) resulted in a significant difference (α ≤ 0.05) in aromas, these compounds may be an important component of the fruity aroma of Qingya-flavored Baijiu. The concentration of ethyl isobutyrate, ethyl caprylate, and ethyl isovalerate was significantly different between two types pf Baijiu, which may be the key to the difference in the intensity of the fruit aroma. The flower aroma of linalool also showed a significant difference, which indicates that linalool may play an important role in the flower aroma of Qingya-flavored Baijiu.

## 4. Conclusions

In this study, 63 compounds with aroma characteristics were qualitatively identified using GC-O combined with GC-MS, and a multivariate method was used for the precise quantification of 61 important aroma compounds. By calculating OAVs with threshold values, 22 compounds were identified as having an impact on the overall aroma. The differences in the types and concentrations of aroma compounds between Qingya-flavored Baijiu and traditional rice-flavored Baijiu were preliminarily analyzed. Static and dynamic sensory experiments helped clarify the sensory characteristics and differences between the two types of Baijiu. Using PLSR, it was possible to predict the compounds related to the characteristic aroma of the two types of Baijiu, and 10 aroma compounds were identified as potential contributors to the differences. (2E,4E)-Deca-2,4-dienal, linalool, apricolin, ethyl acetate, ethyl isobutyrate, ethyl caprylate, and ethyl isovalerate were proven to play a key role in the aroma of Qingya-flavored Baijiu with aroma recombination and omission experiments. Aldehydes may have synergistic effects in the aroma of Qingya-flavored Baijiu. This study provides data support for the direction of innovation and the development of rice-based Baijiu and offers a basis for further research on the sensory properties of these beverages.

## Figures and Tables

**Table 1:** Sample information used in this study.

Sample Name	Types of Baijiu	Region, Country	Company	Alcohol (%vol)
Q40	Qingya-flavored Baijiu	Guangdong, China	A	40
Q50	Qingya-flavored Baijiu	Guangdong, China	A	52
R40-1	Rice-flavored Baijiu	Guangxi, China	B	40
R40-2	Rice-flavored Baijiu	Guangxi, China	C	40
R50-1	Rice-flavored Baijiu	Guangxi, China	B	52
R50-2	Rice-flavored Baijiu	Guangxi, China	C	52

**Table 2:** Aroma compounds identified by GC-O-MS.

Aroma Compound	RI ^a^		RIL		Basis of ID ^b^	Descriptor ^c^	Osme Value					
	DB-FFAP	DB-5	DB-FFAP	DB-5			Q40	R40-1	R40-2	Q50	R50-1	R50-2
ethyl acetate	913	601	981	610	RI, Aroma, MS, Std.	pineapple	3.15	3.12	3.16	3.45	3.06	3.43
ethyl isobutyrate	920	733	960	751	RI, Aroma, MS, Std.	sweet	3.55	3.65	3.65	3.55	3.45	3.62
ethyl butyrate	1021	800	1041	807	RI, Aroma, MS, Std.	apple	2.86	3.1	3.35	3.42	3.54	3.37
ethyl 2-methylbutanoate	1109	844	1073	848	RI, Aroma, MS, Std.	fruit	3.95	3.55	4.36	4.02	4.4	4.5
ethyl isovalerate	1110	856	1080	858	RI, Aroma, MS, Std.	fruit	3.25	3.6	4.56	4.32	4.5	4.66
ethyl valerate	1171	885	1137	904	RI, Aroma, MS, Std.	fruit, green	3.25	3	2.55	-	1.68	1.45
isoamyl acetate	1162	865	1126	881	RI, Aroma, MS, Std.	banana	2.23	2.5	2.2	2.65	2.3	1.96
ethyl hexanoate	1258	989	1220	999	RI, Aroma, MS, Std.	fruit	2.85	2.5	2.86	2.75	2.63	2.9
ethyl lactate	1350	807	1341	815	RI, Aroma, MS, Std.	fruit	0.05	0.25	0.33	0.65	0.3	0.28
ethyl oenanthate	1361	1075	1352	1099	RI, Aroma, MS, Std.	fruit, green	2	2.85	-	-	0.62	-
ethyl caprylate	1435	1188	1435	1197	RI, Aroma, MS, Std.	fruit, fat	2.85	2.6	2.95	2.86	3.01	3.12
ethyl nonanoate	1531	1310	1526	1319	RI, Aroma, MS, Std.	fruit, fat, soap	1.85	3.05	-	-	0.61	-
ethyl DL-leucate	1541	/	1538	/	RI, Aroma, MS, Std.	fruit	3.12	3	2.85	-	2.94	3.17
ethyl benzoate	1677	1166	1652	1187	RI, Aroma, MS, Std.	flower	3.2	3	3.03	3.1	3	3.13
3-methylbutyl octanoate	1695	1436	1670	1450	RI, Aroma, MS, Std.	sweet, fruit	3.05	2.25	1.96	2.46	2.63	2.55
diethyl succinate	1672	1173	1687	1179	RI, Aroma, MS, Std.	wine, fruit	1.85	1.45	-	1.63	2.3	2.26
ethyl undecanoate	1720	1488	1732	1494	RI, Aroma, MS, Std.	coconut	2.7	1.7	2.45	2.36	2.19	2.27
ethyl phenylacetate	1790	1233	1785	1243	RI, Aroma, MS, Std.	Fruit, sweet	2.65	3.25	3.25	3	2.85	3.13
phenylethyl acetate	1823	1249	1825	1260	RI, Aroma, MS, Std.	rose, honey	4.2	3.5	3.65	3.25	3.63	3.33
ethyl laurate	1838	1585	1856	1590	RI, Aroma, MS, Std.	green, fat	1.8	3	-	1.86	2.83	-
ethyl 3-phenylpropanoate	1896	1359	1900	1360	RI, Aroma, MS, Std.	flower	2.6	3.3	3.45	3.19	3.26	3.43
ethyl tetradecanoate	2045	1779	2070	1790	RI, Aroma, MS, Std.	sweet	2.2	2.4	-	-	2.3	2.55
1-propanol	1011	542	1040	568	RI, Aroma, MS, Std.	alcohol, irritation	2.3	3	2.58	2.2	2.74	2.36
isobutanol	1152	639	1093	654	RI, Aroma, MS, Std.	solvent	2.45	1.95	2.45	2.36	2.49	2.21
butanol	1176	645	1150	660	RI, Aroma, MS, Std.	chemical	2.5	2.4	1.9	-	-	1.1
3-methyl-1-butanol	1245	731	1211	732	RI, Aroma, MS, Std.	burnt	4.35	3.8	4.32	4.68	4.25	4.6
1-hexanol	1369	855	1359	869	RI, Aroma, MS, Std.	green	2.7	2	1.88	0.75	1.2	2.03
3-octenol	1442	961	1456	978	RI, Aroma, MS, Std.	mushroom	2.1	1.2	1.39	1.11	1.41	1.08
1-heptanol	1435	958	1470	971	RI, Aroma, MS, Std.	chemical	0.56	1.2	-	-	-	-
1,1-Diethoxyethane	926	720	894	726	RI, Aroma, MS, Std.	Fruit, cream	3.05	3	3.09	3.12	2.95	3.4
hexanal	1105	783	1094	800	RI, Aroma, MS, Std.	grass	4.25	3.5	3.05	3.25	3.16	3.34
(2E)-2-Nonenal	1545	/	1542	/	RI, Aroma, Std.	cucumber	2.2	-	-	2	-	-
(2E,4E)-Deca-2,4-dienal	1772	/	1770	/	RI, Aroma, Std.	fried	1.1	-	-	2.32	-	-
(2E)-2-Octenal	1407	/	1419	/	RI, Aroma, Std.	green, nut	1.5	0.65	-	2.32	1.63	-
trans-2-Undecen-1-al	1762	/	1761	/	RI, Aroma, Std.	green	2.66	-	-	-	-	-
(2E)-2-Decenal	1652	/	1650	/	RI, Aroma, Std.	green	1.2	-	-	-	-	-
nonanal	1405	1094	1400	1104	RI, Aroma, MS, Std.	green, fat	1	0.5	0.36	0.52	0.76	0.85
2-heptanone	1211	884	1213	895	RI, Aroma, MS, Std.	soap	0.65	0.33	-	0.3	-	0.63
acetoin	1307	682	1305	753	RI, Aroma, MS, Std.	butter, cream	3.5	3.6	3.36	3.75	3.13	3.46
2-nonanone	1401	1079	1417	1070	RI, Aroma, MS, Std.	milk, soap	1.25	1.05	-	-	-	-
3-octen-2-one	1421	1025	1414	1036	RI, Aroma, MS, Std.	nut	2.2	1.5	1.2	1.35	1.45	1.67
linalool	1559	1073	1552	1095	RI, Aroma, MS, Std.	flower	4.65	4.1	4.52	4.13	4.62	4.68
cis-anethol	1806	/	1815	/	RI, Aroma, MS, Std.	sweet, spicy	2.3	3.65	-	-	-	-
trans-caryophyllene	1584	1406	1601	1417	RI, Aroma, MS, Std.	wood	1	-	-	0.12	-	0.3
citronellol	1776	1220	1773	1237	RI, Aroma, MS, Std.	rose	2.25	2.64	2.75	2.49	2.81	2.19
geranylacetone	1896	1443	1862	1447	RI, Aroma, MS, Std.	green	1.46	1.22	-	-	-	-
acetic acid	1476	612	1468	600	RI, Aroma, MS, Std.	sour	4.15	4.5	4.55	4.65	4.51	4.35
isobutyric acid	1552	749	1552	775	RI, Aroma, MS, Std.	cheese	4.55	3.35	3.85	4.1	3.94	3.76
2-methylbutyric acid	1662	857	1688	868	RI, Aroma, MS, Std.	cheese	4.5	4	3.68	3.89	4.21	4.3
octanoic acid	2075	1185	2050	1166	RI, Aroma, MS, Std.	cheese, sweaty	2.1	2.6	2.13	2.06	2.45	2.65
2-amylfuran	1257	982	1261	991	RI, Aroma, MS, Std.	butter	1.05	2.65	0.52	-	0.86	1.27
furfural	1475	826	1482	826	RI, Aroma, MS, Std.	sweet, nut, wood	3.25	3.05	3.2	3.12	2.35	3.03
styrene	1286	902	1275	889	RI, Aroma, MS, Std.	gasoline, balm	1.05	2	1.34	0.35	1.3	0.6
acetophenone	1668	1054	1664	1065	RI, Aroma, MS, Std.	flower, almond	2.25	3.15	2.85	2.95	2.77	2.42
benzyl alcohol	1882	1020	1905	1031	RI, Aroma, MS, Std.	Sweet, flower	1.5	2.55	1.96	1.78	1.66	1.2
phenethyl alcohol	1923	1110	1899	1120	RI, Aroma, MS, Std.	flower, honey	4.65	4.6	4.85	4.69	4.82	4.67
apricolin	2053	1362	2056	1363	RI, Aroma, MS, Std.	coconut, peach	3.25	4.6	3.92	3.75	4.06	3.87
1-hexanoic acid	1835	985	1860	991	RI, Aroma, MS, Std.	sour, cheese	4.25	4.12	3.88	3.65	3.95	3.01
propionic acid	1530	706	1557	693	RI, Aroma, MS, Std.	sour	4.02	4.01	3.85	3.74	3.68	3.9
benzaldehyde	1520	975	1505	964	RI, Aroma, MS, Std.	almond, flower	2.52	1.05	-	2.26	0.94	0.42
4-ethylphenol	2215	1165	2202	1171	RI, Aroma, MS, Std.	phenol, spice	3.2	3.25	2.95	3.01	3.2	3.02
unknown	1313	/	/	/	Aroma	cream, chocolate	2.86	2.5	-	3.2	3.05	-
unknown	1775	/	/	/	Aroma	cooked vegetable	4.52	4.62	4.75	4.56	4.65	4.85

^a^ Retention index determined by GC-MS on two different stationary phases (DB-FFAP and DB-5). ^b^ The basis for qualitative compounds, including RI (retention index), aroma (odor description by comparison to the reference standards by GC-O), MS (mass spectrometry), and Std. (standards). ^c^ Aroma of compounds detected during GC-O process. “/”, not determined. “-“, not detected at the sniffing port.

**Table 3:** The odor activity values (OAVs) of aroma compounds.

Aroma Compound	Threshold (μg/L)	OAVs					
		Q40	R40-1	R40-2	Q50	R50-1	R50-2
ethyl caprylate	12.9 ^a^	67.57	126.09	71.06	124.84	146.90	190.58
ethyl isobutyrate	57.5 ^a^	18.78	85.83	64.86	9.23	56.75	102.74
ethyl hexanoate	55.3 ^a^	8.52	7.46	4.04	8.49	8.21	6.79
ethyl isovalerate	6.89 ^b^	14.64	42.10	39.74	13.00	47.64	65.41
hexanal	25.5 ^a^	27.00	2.34	1.72	9.55	3.65	4.86
3-octenol	6.12 ^a^	12.51	12.81	5.83	6.82	20.00	13.75
ethyl Dl-leucate	57 ^c^	48.41	66.02	34.25	35.84	71.87	189.28
ethyl butyrate	81.5 ^a^	8.41	15.94	5.65	9.07	12.39	9.63
1-hexanoic acid	2520 ^a^	6.16	<1	1.08	5.19	<1	<1
ethyl 2-methylbutanoate	18 ^d^	4.57	16.24	7.68	4.19	16.20	15.20
ethyl valerate	26.8 ^a^	6.66	4.93	<1	6.13	2.31	1.90
(2E)-2-Octenal	15.1 ^d^	17.96	10.44	<1	7.94	11.97	<1
(2E,4E)-Deca-2,4-dienal	7.71 ^d^	13.89	<1	1.96	39.35	3.24	2.16
isobutanol	28,300 ^e^	12.12	4.74	12.80	15.84	20.47	15.27
ethyl acetate	32,600 ^a^	7.78	4.65	12.28	7.70	4.88	14.59
ethyl laurate	400 ^b^	1.22	<1	<1	1.96	1.92	2.23
2-methylbutyric acid	5931 ^d^	1.18	<1	<1	<1	1.22	<1
isoamyl acetate	94 ^b^	6.25	9.80	6.93	8.26	17.35	20.48
1-propanol	54,000 ^d^	3.72	2.02	2.02	3.89	2.95	3.25
butanol	2730 ^e^	3.08	4.27	1.37	4.08	3.92	5.68
linalool	13.1 ^f^	1.94	3.48	1.48	2.20	<1	1.66
apricolin	90.7 ^a^	1.76	<1	<1	1.80	<1	<1
ethyl lactate	128,000 ^a^	2.85	3.06	2.90	2.81	1.98	4.04
(2E)-2-Decenal	12.1 ^d^	4.65	<1	1.51	<1	1.19	<1
phenethyl alcohol	28,900 ^b^	<1	1.64	1.31	<1	1.76	1.78
isobutyric acid	2300 ^c^	<1	<1	<1	<1	1.16	<1
(2E)-2-Nonenal	50.5 ^d^	<1	<1	<1	<1	<1	<1
nonanal	122 ^a^	<1	<1	<1	<1	<1	<1
2-amylfuran	100 ^c^	<1	<1	<1	<1	<1	<1
1-hexanol	5370 ^a^	<1	<1	<1	<1	<1	<1
octanoic acid	2700 ^d^	<1	<1	<1	<1	<1	<1
cis-anethol	50 ^c^	<1	<1	<1	<1	<1	<1
ethyl phenylacetate	407 ^a^	<1	<1	<1	<1	<1	<1
phenylethyl acetate	909 ^a^	<1	<1	<1	<1	<1	<1
furfural	44,000 ^a^	<1	<1	<1	<1	<1	<1
acetophenone	256 ^a^	<1	<1	<1	<1	<1	<1
acetic acid	200,000 ^a^	<1	<1	<1	<1	<1	<1
3-methylbutyl octanoate	125 ^c^	<1	<1	<1	<1	<1	<1
3-methyl-1-butanol	179,190 ^e^	<1	1.06	1.50	<1	<1	<1
ethyl 3-phenylpropanoate	130 ^b^	<1	<1	<1	<1	<1	<1
acetoin	259 ^d^	<1	<1	1.36	<1	<1	2.31
1,1-Diethoxyethane	2090 ^a^	<1	<1	<1	<1	<1	<1
trans-caryophyllene	64 ^c^	<1	<1	<1	<1	<1	<1
propionic acid	18,200 ^b^	<1	<1	<1	<1	<1	<1
2-heptanone	680 ^c^	<1	<1	<1	<1	<1	<1
citronellol	100 ^c^	<1	<1	<1	<1	<1	<1
ethyl benzoate	1400 ^a^	<1	<1	<1	<1	<1	<1
trans-2-Undecen-1-al	240 ^d^	<1	<1	<1	<1	<1	<1
3-octen-2-one	250 ^c^	<1	<1	<1	<1	<1	<1
ethyl nonanoate	3200 ^a^	<1	<1	<1	<1	<1	<1
2-nonanone	483 ^e^	<1	<1	<1	<1	<1	<1
geranylacetone	267 ^b^	<1	<1	<1	<1	<1	<1
diethyl succinate	353,000 ^a^	<1	<1	<1	<1	<1	<1
styrene	1400 ^d^	<1	<1	<1	<1	<1	<1
benzyl alcohol	40,900 ^b^	<1	<1	<1	<1	<1	<1
1-heptanol	26,600 ^a^	<1	<1	<1	<1	<1	<1
ethyl undecanoate	1000 ^c^	<1	<1	<1	<1	<1	<1
ethyl oenanthate	13,200 ^a^	<1	<1	<1	<1	<1	<1
benzaldehyde	4203.1 ^e^	<1	<1	<1	<1	<1	<1
4-ethylphenol	617.68 ^e^	<1	<1	<1	<1	<1	<1
ethyl tetradecanoate	494,000 ^e^	<1	<1	<1	<1	<1	<1

^a^ Odor thresholds were obtained from reference [29]. ^b^ Odor thresholds were obtained from reference [30]. ^c^ Odor thresholds were obtained from www.thresholdcompilation.com. URL (accessed on 1 May 2022) ^d^ Odor thresholds were obtained from reference [10]. ^e^ Odor thresholds were obtained from reference [12]. ^f^ Odor thresholds were obtained from reference [31].

## Data Availability

The original contributions presented in the study are included in the article/Supplementary Material, further inquiries can be directed to the corresponding author.

## Supplementary Materials

The following supporting information can be downloaded at: https://www.mdpi.com/article/10.3390/foods13050681/s1, Table S1: Compound Quantitative Standard Curve Information Table; Table S2: Specific Explanation and Reference of Sensory Descriptors; Table S3: Concentrations of aroma compounds.

## References

[1] Jin G., Zhu Y., Xu Y. (2017). Mystery behind Chinese liquor fermentation. Trends Food Sci. Technol..

[2] Fan W.L., Qian M.C. (2006). Characterization of aroma compounds of Chinese “Wuliangye” and “Jiannanchun” liquors by aroma extract dilution analysis. J. Agric. Food Chem..

[3] Carrau F., Gaggero C., Aguilar P.S. (2015). Yeast diversity and native vigor for flavor phenotypes. Trends Biotechnol..

[4] Wei Y., Zou W., Shen C., Yang J. (2020). Basic flavor types and component characteristics of Chinese traditional liquors: A review. J. Food Sci..

[5] Fang C., Du H., Jia W., Xu Y. (2018). Compositional Differences and Similarities between Typical Chinese Baijiu and Western Liquor as Revealed by Mass Spectrometry-Based Metabolomics. Metabolites.

[6] Li Z., Wang N., Raghavan G.S.V., Vigneault C. (2008). Volatiles Evaluation and Dielectric Properties Measurements of Chinese Spirits for Quality Assessment. Food Bioprocess Technol..

[7] Wu J.-F., Xu Y. (2013). Comparison of Pyrazine Compounds in Seven Chinese Liquors Using Headspace Solid-phase Micro-extraction and GC-Nitrogen Phosphourus Detection. Food Sci. Biotechnol..

[8] Zhu S.K., Lu X., Ji K.H., Guo K.F., Li Y.L., Wu C.Y., Xu G. (2007). Characterization of flavor compounds in Chinese liquor Moutai by comprehensive two-dimensional gas chromatography/time-of-flight mass spectrometry. Anal. Chim. Acta.

[9] Yin X., Yoshizaki Y., Kurazono S., Sugimachi M., Takeuchi H., Han X.-L., Okutsu K., Futagami T., Tamaki H., Takamine K. (2020). Characterization of Flavor Compounds in Rice-flavor baijiu, a Traditional Chinese Distilled Liquor, Compared with Japanese Distilled Liquors, awamori and kome-shochu. Food Sci. Technol. Res..

[10] Fan H.Y., Fan W.L., Xu Y. (2015). Characterization of Key Odorants in Chinese Chixiang Aroma-Type Liquor by Gas Chromatography-Olfactometry, Quantitative Measurements, Aroma Recombination, and Omission Studies. J. Agric. Food Chem..

[11] He Y., Liu Z., Qian M., Yu X., Xu Y., Chen S. (2020). Unraveling the chemosensory characteristics of strong-aroma type Baijiu from different regions using comprehensive two-dimensional gas chromatography-time-of-flight mass spectrometry and descriptive sensory analysis. Food Chem..

[12] Li H., Zhang X., Gao X., Shi X., Chen S., Xu Y., Tang K. (2023). Comparison of the Aroma-Active Compounds and Sensory Characteristics of Different Grades of Light-Flavor Baijiu. Foods.

[13] Zhao Z., Sugimachi M., Yoshizaki Y., Yin X., Han X.-L., Okutsu K., Futagami T., Tamaki H., Takamine K. (2021). Correlation between key aroma and manufacturing processes of rice-flavor baijiu and awamori, Chinese and Japanese traditional liquors. Food Biosci..

[14] He Y., Chen S., Tang K., Qian M., Yu X., Xu Y. (2021). Sensory characterization of Baijiu pungency by combined time-intensity (TI) and temporal dominance of sensations (TDS). Food Res. Int..

[15] Wang J.S., Chen H., Wu Y.S., Zhao D.R. (2022). Uncover the flavor code of strong-aroma baijiu: Research progress on the revelation of aroma compounds in strong-aroma baijiu by means of modern separation technology and molecular sensory evaluation. J. Food Compos. Anal..

[16] Yan Y., Chen S., Nie Y., Xu Y. (2020). Characterization of volatile sulfur compounds in soy sauce aroma type Baijiu and changes during fermentation by GC x GC-TOFMS, organoleptic impact evaluation, and multivariate data analysis. Food Res. Int..

[17] Fan S., Tang K., Xu Y., Chen S. (2020). Characterization of the potent odorants in Tibetan Qingke Jiu by sensory analysis, aroma extract dilution analysis, quantitative analysis and odor activity values. Food Res. Int..

[18] Huang L., Ma Y., Tian X., Li J.-M., Li L.-X., Tang K., Xu Y. (2018). Chemosensory characteristics of regional Vidal icewines from China and Canada. Food Chem. Food Chem..

[19] Xu Y., Zhao J., Liu X., Zhang C., Zhao Z., Li X., Sun B. (2021). Flavor mystery of Chinese traditional fermented baijiu: The great contribution of ester compounds. Food Chem..

[20] Mu Y., Huang J., Zhou R., Zhang S., Qin H., Tang H., Pan Q., Tang H. (2023). Characterization of the differences in aroma-active compounds in strong-flavor Baijiu induced by bioaugmented Daqu using metabolomics and sensomics approaches. Food Chem..

[21] Yu H., Li Q., Xie J., Chen C., Lou X., Ai L., Tian H. (2022). Characterization of Bitter Compounds in Shaoxing Huangjiu by Quantitative Measurements, Taste Recombination, and Omission Experiments. J. Agric. Food Chem..

[22] Hong J.X., Zhao D.R., Sun B.G. (2021). Research Progress on the Profile of Trace Components in Baijiu. Food Rev. Int..

[23] Yoshizaki Y., Yamato H., Takamine K., Tamaki H., Ito K., Sameshima Y. (2010). Analysis of Volatile Compounds in Shochu Koji, Sake Koji, and Steamed Rice by Gas Chromatography-Mass Spectrometry. J. Inst. Brew..

[24] Wu Y., Hou Y., Chen H., Wang J., Zhang C., Zhao Z., Ao R., Huang H., Hong J., Zhao D. (2022). “Key Factor” for Baijiu Quality: Research Progress on Acid Substances in Baijiu. Foods.

[25] Li Y., Li Q., Zhang B., Shen C., Xu Y., Tang K. (2021). Identification, quantitation and sensorial contribution of lactones in brandies between China and France. Food Chem..

[26] Liu S.P., Yang Q.L., Mao J.Q., Bai M., Zhou J.D., Han X., Mao J. (2020). Feedback inhibition of the prephenate dehydratase from Saccharomyces cerevisiae and its mutation in huangjiu (Chinese rice wine) yeast. Lwt-Food Sci. Technol..

[27] Jezussek M., Juliano B.O., Schieberle P. (2002). Comparison of key aroma compounds in cooked brown rice varieties based on aroma extract dilution analyses. J. Agric. Food Chem..

[28] Rahayu Y.Y.S., Yoshizaki Y., Yamaguchi K., Okutsu K., Futagami T., Tamaki H., Sameshima Y., Takamine K. (2017). Key volatile compounds in red koji-shochu, a Monascus-fermented product, and their formation steps during fermentation. Food Chem..

[29] Gao W.J., Fan W.L., Xu Y. (2014). Characterization of the Key Odorants in Light Aroma Type Chinese Liquor by Gas Chromatography-Olfactometry, Quantitative Measurements, Aroma Recombination, and Omission Studies. J. Agric. Food Chem..

[30] Chen S., Tang J., Fan S., Zhang J., Chen S., Liu Y., Yang Q., Xu Y. (2021). Comparison of Potent Odorants in Traditional and Modern Types of Chinese Xiaoqu Liquor (Baijiu) Based on Odor Activity Values and Multivariate Analyses. Foods.

[31] Wang L., Hu G., Lei L., Lin L., Wang D., Wu J. (2015). Identification and Aroma Impact of Volatile Terpenes in Moutai Liquor. Int. J. Food Prop..

[32] Pu D.D., Duan W., Huang Y., Zhang L.L., Zhang Y.Y., Sun B.G., Ren F.Z., Zhang H.Y., Tang Y.Z. (2020). Characterization of the dynamic texture perception and the impact factors on the bolus texture changes during oral processing. Food Chem..

