# Supplementary material for: Comparison of Aroma Compounds and Sensory Characteristics between Two Different Types of Rice-Based Baijiu
Source: Foods. 2024 Feb 23;13(5):681. doi: 10.3390/foods13050681 (PMC10930833; doi:10.3390/foods13050681)
Supplement: Supplementary file 1 [file foods-13-00681-s001.zip › foods-2872102-supplementary.pdf]

Table S1. Compound Quantitative Standard Curve Information Table.

Compound	Method	Retention Time	Quantitative Ion (m/z)	Standard Curve	R^2	Concentration Range(μg/L)
ethyl acetate	GC-FID	7.49	-	y=0.9096x+0.0315	0.9996	262.144-66440.000*
1-propanol	GC-FID	12.795	-	y=0.9921x+0.006	0.9995	26.590-851.030*
isobutanol	GC-FID	14.952	-	y=0.854x+0.0053	0.9995	9.380-300.010*
3-methyl-1-butanol	GC-FID	19.085	-	y=0.7092x+0.0084	0.9994	13.460-430.670*
ethyl lactate	GC-FID	23.66	-	y=1.1307x+0.0689	0.9966	71.690-2293.990*
1,1-Diethoxyethane	SPME-GC-MS	6.274	45	y=23.189x-94.988	0.9935	21.703-1388.88
ethyl isobutyrate	SPME-GC-MS	7.426	71	y=87.562x+2.9627	0.9945	26.67-6828.27
ethyl butyrate	SPME-GC-MS	8.555	71	y=49.301x+1.7781	0.9986	513.85-6577
ethyl 2-methylbutanoate	SPME-GC-MS	8.992	102	y=24.136x+0.2673	0.9985	0.416-425.9
ethyl isovalerate	SPME-GC-MS	9.14	88	y=18.398x+0.3021	0.9936	4.134-1058
hexanal	SPME-GC-MS	9.47	56	y=1.0154x-0.134	0.9976	67.51-17284
ethyl valerate	SPME-GC-MS	10.41	57	y=49.72x-0.0317	0.996	3.9-40038
butanol	SPME-GC-MS	11.125	56	y=12.853x+2.417	0.9923	162.41-83153.4
ethyl hexanoate	SPME-GC-MS	12.513	88	y=14.936x+0.2718	0.9986	33.63-68105
acetoin	SPME-GC-MS	15.25	45	y=0.2747x-0.0086	0.9921	20.59-21090
styrene	SPME-GC-MS	13.3	104	y=0.2663x-0.2411	0.9907	4.88-50000
ethyl oenanthate	SPME-GC-MS	14.76	88	y=4.2755x+0.1113	0.9936	24.41-6250
1-hexanol	SPME-GC-MS	15.421	55	y=25.427x-2.6501	0.9954	196.74-201465
2-nonanone	SPME-GC-MS	16.197	58	y=0.2855x-0.066	0.9988	7.436-3807.5
nonanal	SPME-GC-MS	16.269	98	y=2.2655x-0.1561	0.9939	7.79-1998.6
3-octen-2-one	SPME-GC-MS	16.676	111	y=6.6114x-0.0041	0.9995	15.625-1000
ethyl caprylate	SPME-GC-MS	17.159	88	y=4.5887x-1.9988	0.9987	37.65-19277
1-Heptanol	SPME-GC-MS	17.539	70	y=3.4364x-0.0214	0.9974	25.56-13089
furfural	SPME-GC-MS	18.174	96	y=59.6x+8.2453	0.9949	82.95-21237

ethyl nonanoate	SPME-GC-MS	19.212	88	y=2.1613x-0.1251	0.9964	17.05-8727.75
linalool	SPME-GC-MS	19.4	121	y=2.4273x-1.2006	0.9996	3.8-1950
trans-caryophyllene	SPME-GC-MS	20.619	93	y=0.2977x+0.0408	0.9968	2.008-257.025
ethyl benzoate	SPME-GC-MS	22.17	105	y=5.0435x-0.1309	0.9965	5.8-2969.5
3-methylbutyl octanoate	SPME-GC-MS	21.717	127	y=3.6608x+0.0092	0.9961	20.471-5241
diethyl succinate	SPME-GC-MS	22.086	101	y=145.25x-1.4796	0.9939	46.03-23569
ethyl undecanoate	SPME-GC-MS	23.238	88	y=0.8464x+0.0142	0.9922	0.416-425.9
citronellol	SPME-GC-MS	23.75	69	y=0.174x+0.004	0.9998	7.47-956.37
ethyl phenylacetate	SPME-GC-MS	24.264	91	y=5.289x+0.0025	9.9966	22.07-5813.5
cis-anethol	SPME-GC-MS	25.444	104	y=0.6408x-25.487	0.9913	24.41-6250
phenylethyl acetate	SPME-GC-MS	24.845	104	y=6.0501x+0.1366	0.9939	22.07-5813.5
ethyl laurate	SPME-GC-MS	25.128	88	y=1.755x-0.0538	0.9978	8.64-4425
benzyl alcohol	SPME-GC-MS	25.877	79	y=118.7x+8.5064	0.9991	107.48-55030
ethyl 3-phenylpropanoate	SPME-GC-MS	26.048	104	y=3.5418x+0.0778	0.9946	14.94-7649
phenethyl alcohol	SPME-GC-MS	26.538	91	y=18.201x-2.1329	0.9982	98.43-50400
2-heptanone	SPME-GC-MS	11.559	58	y = 0.8688x - 0.0239	0.9932	1.54-792.4
apricolin	SPME-GC-MS	28.591	85	y=1.6942x+0.009	0.9963	3.57-1830
ethyl tetradecanoate	SPME-GC-MS	28.5	88	y=1.7726x+0.0619	0.9959	19.53-10000
geranylacetone	SPME-GC-MS	25.489	69	y=0.1756x-0.0226	0.9925	3.47-888
2-amylfuran	SPME-GC-MS	12.277	81	y=0.3848x+0.2014	0.9992	1.95-2000
3-octenol	SPME-GC-MS	17.402	57	y=1.0232x+0.0277	0.9988	14.02-1794
ethyl Dl-leucate	SPME-GC-MS	19.45	104	y=1746.4x+0.3136	0.9983	15.5-7950
acetophenone	SPME-GC-MS	21.911	105	Y=13.258x-4.589	0.9909	2.28-585
isobutyric acid	SPME-GC-MS	20.086	88	y=506.37x+13.015	0.9973	2.86718-367*
2-methylbutyric acid	SPME-GC-MS	22.059	60	y=860.94x+0.7899	0.9969	2.13-273.80*

isoamyl acetate	SPME-GC-MS	10.23	70	y=0.1823x-0.5192	0.9954	19.53-10000
benzaldehyde	SPME-GC-MS	19.509	106	y = 1.3913x - 0.4087	0.9942	5.85-749.86
4-ethylphenol	SPME-GC-MS	30.679	107	y = 3.5258x - 0.4433	0.9984	6.21-3182.19
octanoic acid	LLME-GC-MS	29.306	73	y=168.38x+19.605	0.9949	2.35-301.40*
propionic acid	LLME-GC-MS	19.687	57	y=404.99x-0.278	0.9964	122.26-15650
1-hexanoic acid	LLME-GC-MS	25.162	60	y=94.386x+21.629	0.995	2.81-360.80*
acetic acid	LLME-GC-MS	17.885	60	y=43.849x+16.104	0.9975	1.65-211.760*
(2E)-2-Nonenal	SPME-GC×GC-TOF MS	-	83	y=11.970x+0.0345	0.9931	4.19-134.20
(2E,4E)-Deca-2,4-dienal	SPME-GC×GC-TOF MS	-	81	y=7.7726x+0.0152	0.9973	3.57-57.125
(2E)-2-Octenal	SPME-GC×GC-TOF MS	-	83	y=24.552x-0.0408	0.9948	3.91-2000
trans-2-Undecen-1-al	SPME-GC×GC-TOF MS	-	70	y=1.5511x-0.0078	0.9963	3.91-500
(2E)-2-Decenal	SPME-GC×GC-TOF MS	-	70	y=1.6879x-0.1002	0.9957	3.48-445

* The concentration unit is mg/L. - Determine based on RI.

Table S2. Specific Explanation and Reference of Sensory Descriptors.

Number	Descriptor	Descriptor Definition	Reference	Score*
1	Fruity aroma	Aroma of ripe fruit	10 mg/L ethyl hexanoate and 5 mg/L isoamyl acetate in 40% ethanol aqueous solution	9
2	Flower aroma	Floral-like aroma	10 mg/L phenylethyl acetate and 10 mg/L β-phenylethyl alcohol in 40% ethanol aqueous solution	9
3	Plant aroma	Plant-like aromas such as trees and herbs	Le Nez du Vin Cedor and Green Pepper	9
4	Roasted aroma	An aroma similar to the smell of toasting bread.	Real toast	9
5	Oily aroma	Oil-like aroma	Chi-flavored Baijiu	9
6	Sweet aroma	Suger-like and Honey-like aroma	250 μg/L β-damascenone and 100 μg/L apricolin in 40% ethanol aqueous solution	9
7	Rice aroma	Aroma similar to the taste of cooked rice	Cooked rice	9
8	Sour aroma	Vinegar-like aroma	Real vinegar	9
9	Sweetness	Sweetness in Baijiu	10 g/L edible sucrose in 40% ethanol aqueous solution	-
10	Stimulation	Burning sensation when drinking Baijiu	40% ethanol aqueous solution	-
11	Bitterness	Bitterness of Baijiu	Bitter gourd juice	-

* Maximum intensity value of sensory descriptors. - No sensory intensity values.

Table S3. Concentrations of aroma compounds.

Aroma compound	Concentration (μg/L)						Statistical significance[a]	
	Q40	R40-1	R40-2	Q50	R50-1	R50-2	Group 40[b]	Group 52[c]
Esters								
ethyl lactate	364172.64±0.01	391353.97±0.01	371178.82±0.01	359458.78±0.01	252843.25±0.01	517494.43±0.00	-	-
ethyl acetate	253492.78±0.01	151613.84±0.09	400313.54±0.03	250940.30±0.00	158992.29±0.10	475677.91±0.03	-	-
diethyl succinate	4631.55±0.20	6520.46±0.15	6615.88±0.15	4992.04±0.47	11541.66±0.17	15309.63±0.13	-	*
ethyl isobutyrate	1079.91±0.33	4934.96±0.06	3729.72±0.07	530.90±0.32	3263.12±0.20	5907.72±0.11	*	*
ethyl hexanoate	471.31±0.08	412.50±0.06	223.54±0.11	469.42±0.05	453.87±0.05	375.66±0.06	-	-
ethyl caprylate	871.71±0.08	1626.57±0.04	916.65±0.07	1610.48±0.11	1895.05±0.33	2458.44±0.26	-	-
ethyl butyrate	685.73±0.16	1299.11±0.02	460.45±0.05	738.92±0.11	1010.07±0.23	784.52±0.29	*	-
ethyl Dl-leucate	2759.27±0.04	3763.32±0.02	1952.32±0.04	2042.97±0.07	4096.85±0.97	10789.18±0.37	*	-
ethyl laurate	486.19±0.01	58.79±0.58	393.99±0.09	785.50±0.06	768.82±0.25	890.90±0.22	*	-
ethyl tetradecanoate	352.66±0.01	35.26±0.71	226.84±0.11	1217.76±0.12	1049.42±0.11	1244.87±0.09	*	-
ethyl valerate	178.40±0.02	132.22±0.01	14.12±0.11	164.40±0.05	61.84±0.06	50.97±0.07	*	*
ethyl isovalerate	100.90±0.20	290.08±0.05	273.82±0.06	89.55±0.81	328.24±0.24	450.68±0.18	*	*
ethyl nonanoate	95.15±0.02	12.84±0.00	0.05±0.60	145.69±0.03	34.52±0.09	20.70±0.15	*	*
isoamyl acetate	587.60±0.37	921.63±0.18	651.55±0.26	776.80±0.23	1631.31±0.23	1924.70±0.19	-	*
ethyl 2-methylbutanoate	82.33±0.14	292.31±0.03	138.16±0.06	75.51±0.57	291.51±0.12	273.64±0.13	*	*
ethyl benzoate	68.24±0.00	1.42±1.42	6.55±0.31	80.41±0.02	12.87±0.18	16.28±0.14	*	*
ethyl oenanthate	63.51±0.01	29.27±0.10	16.26±0.19	54.32±0.04	29.47±0.11	25.69±0.12	*	*

3-methylbutyl octanoate	14.16±0.08	8.23±0.32	12.73±0.20	30.67±0.11	33.09±0.15	39.30±0.13	-	-
ethyl 3-phenylpropanoate	13.76±0.14	10.49±0.07	8.85±0.09	17.44±0.10	14.51±0.14	23.47±0.09	-	-
ethyl undecanoate	6.08±0.02	2.12±0.19	2.57±0.16	10.72±0.25	7.17±0.10	5.35±0.13	*	*
Alcohols								
isobutanol	343119.22±0.00	134259.39±0.04	362224.79±0.02	448309.47±0.05	579391.00±0.28	432160.52±0.37	-	-
1-propanol	200727.63±0.02	109087.05±0.03	109097.17±0.03	210167.50±0.02	159222.88±0.08	175486.26±0.07	*	*
3-methyl-1-butanol	102882.35±0.05	189912.34±0.04	267994.52±0.03	144656.84±0.04	162692.15±0.02	122309.29±0.03	-	-
butanol	8402.35±0.25	11665.58±0.06	3749.91±0.17	11146.38±0.09	10715.05±0.16	15496.95±0.11	*	-
1-hexanol	994.87±0.13	442.72±0.10	272.57±0.17	1470.82±0.02	395.38±0.04	320.49±0.04	*	*
1-heptanol	167.46±0.06	53.14±0.01	6.65±0.10	200.83±0.03	61.57±0.14	120.25±0.07	*	*
3-octenol	76.55±0.02	78.40±0.04	35.68±0.09	41.77±0.14	122.39±0.08	84.17±0.11	-	*
Aldehydes								
1,1-Diethoxyethane	976.41±0.05	393.55±0.17	1074.30±0.06	1000.84±0.14	1078.64±0.06	1105.97±0.06	-	-
hexanal	688.39±0.02	59.62±0.10	43.80±0.13	243.52±0.06	93.07±0.32	123.95±0.24	*	*
nonanal	94.93±0.03	13.94±0.05	9.76±0.07	41.39±0.08	27.87±0.30	28.74±0.29	*	*
(2E)-2-Nonenal	41.02±0.00	19.14±0.00	0.00±0.00	26.96±0.00	0.00±0.00	0.00±0.00	*	*
(2E,4E)-Deca-2,4-dienal	107.10±0.00	3.24±0.39	15.09±0.08	303.35±0.00	24.99±0.03	16.65±0.05	*	*
(2E)-2-Octenal	271.25±0.02	157.64±0.00	14.54±0.03	119.96±0.66	180.79±0.00	0.00±0.00	*	-
trans-2-Undecen-1-al	64.65±0.00	0.00±0.00	16.65±0.04	5.94±0.29	14.14±0.00	0.00±0.00	*	*
(2E)-2-Decenal	56.31±0.00	0.00±0.00	18.33±0.08	0.00±0.00	14.35±0.03	11.85±0.04	*	*

Ketones								
acetoin	136.71±0.08	117.62±0.19	352.93±0.06	188.34±0.06	143.70±0.41	598.95±0.10	-	-
3-octen-2-one	52.94±0.00	0.00±0.00	0.00±0.00	51.99±0.00	0.00±0.00	0.00±0.00	*	*
2-heptanone	52.64±0.00	7.31±0.02	4.02±0.03	11.99±0.41	10.43±0.08	10.30±0.08	*	-
2-nonanone	9.76±0.00	0.00±0.00	0.00±0.00	1.43±0.00	0.00±0.00	0.00±0.00	*	*
Acids								
acetic acid	126317.48±0.01	68044.44±0.14	162072.93±0.06	134998.52±0.07	105664.42±0.07	147458.99±0.05	-	-
1-hexanoic acid	15530.95±0.00	854.39±0.24	2720.32±0.07	13087.64±0.00	826.50±0.03	717.73±0.03	-	*
2-methylbutyric acid	7011.18±0.03	5896.63±0.02	4860.16±0.03	3866.02±0.01	7236.08±0.00	4904.07±0.00	*	*
propionic acid	1745.73±0.02	1232.59±0.02	429.25±0.06	3784.20±0.02	3343.86±0.00	4610.24±0.00	*	*
isobutyric acid	1740.04±0.13	1967.03±0.03	1332.24±0.04	1537.98±0.06	2673.75±0.08	1933.07±0.11	-	-
octanoic acid	1302.98±0.06	976.36±0.02	1190.27±0.02	935.92±0.01	883.38±0.05	1290.53±0.04	-	-
Terpenes								
cis-anethol	4.23±0.00	0.00±0.00	0.00±0.00	40.92±0.00	0.00±0.00	0.00±0	*	*
citronellol	27.12±0.14	21.12±0.49	28.38±0.36	34.74±0.05	33.97±0.07	27.46±0.09	-	-
linalool	25.46±0.08	45.57±0.14	19.41±0.32	28.83±0.03	9.75±0.35	21.70±0.16	-	*
trans-caryophyllene	5.78±0.03	3.98±0.40	9.11±0.18	8.63±0.17	10.31±0.48	12.94±0.38	-	-
geranylacetone	5.39±0.02	0.83±0.50	0.87±0.48	5.24±0.32	4.10±0.36	7.38±0.20	*	-
Aromatic								
phenethyl alcohol	28074.17±1.25	47438.75±0.59	37999.53±0.74	19334.32±1.26	50933.12±0.09	51562.43±0.09	-	*
benzyl alcohol	58.46±0.01	26.08±0.05	26.19±0.05	61.64±0.04	36.29±0.08	33.43±0.08	*	*
acetophenone	46.82±0.01	5.86±0.15	21.30±0.04	32.35±0.07	25.46±0.16	18.05±0.22	-	*
styrene	13.54±0.00	0.00±0.00	0.00±0.00	78.30±0.00	0.00±0.00	0.00±0.00	*	*
benzaldehyde	127.89±0.02	7.30±0.27	17.86±0.11	153.14±0.01	53.35±0.08	72.73±0.06	*	*
4-ethylphenol	24.84±0.21	142.02±0.02	6.37±0.48	46.89±0.09	232.95±0.03	41.62±0.15	*	-

phenylethyl acetate	107.21±0.12	70.60±0.21	74.79±0.20	158.19±0.31	201.24±0.10	128.67±0.16	*	-
ethyl phenylacetate	57.67±0.07	20.16±1.16	256.45±0.09	70.20±0.24	66.04±0.33	112.05±0.20	*	-
Others								
furfural	1221.50±0.03	512.96±0.06	566.66±0.05	1461.55±0.07	645.23±0.06	1349.59±0.03	*	-
apricolin	159.78±0.07	81.13±0.00	56.56±0.00	163.67±0.04	88.49±0.03	33.53±0.07	*	*
2-amylfuran	95.48±0.00	19.45±0.01	24.45±0.01	60.92±0.12	43.44±0.29	29.60±0.42	*	*

[a] Differences in substance concentration between two groups of Qingya-flavored Baijiu and traditional Rice-flavored Baijiu. [b] Team of Q40, R40-1 and R40-2. [c] Team of Q50, R50-1 and R50-2. * Substances with statistical significance. - Substances without statistical significance.
